# Effects of Storage and Roasting Condition on the Antioxidant Activity of Soybeans with Different Colors of Seed Coat

**DOI:** 10.3390/foods12010092

**Published:** 2022-12-24

**Authors:** Takako Koriyama, Kiriko Teranaka, Mitose Tsuchida, Midori Kasai

**Affiliations:** 1 Faculty of Food and Nutritional Science, Toyo University, 1-1-1 Izumino, Itakura-machi, Ora-gun 374-0193, Japan; 2 Department of Nutrition and Food Science, Ochanomizu University, 2-1-1 Otsuka, Bunkyo-ku, Tokyo 112-8610, Japan; 3 Faculty of Health and Nutrition, Bunkyo University, 1100 Namegaya, Chigasaki-shi 253-8550, Japan

**Keywords:** roasting, storage, soybean, seed coat color, aged beans, hard-to-cook, antioxidant activity

## Abstract

The DPPH radical scavenging activity and ORAC value of soybeans (yellow soybean, blue soybean, and black soybean) were increased by roasting at above 190 °C. Concerning raw beans, black soybeans with the darkest seed coat color had the strongest antioxidant activity, indicating the effect of the coat pigment. However, the degree of increased antioxidant activity by roasting was almost similar regardless of seed coat color, suggesting that coat color is independent of the increased antioxidant activity. Concerning aged beans stored at 37 °C/75% RH for 60 days, the antioxidant activity increased in yellow soybean and decreased in blue and black soybean compared to before storage. Conversely, when roasted at 190 °C for 20 min, the DPPH values of all the aged beans were significantly increased. Other analyses of roasted beans with and without seed coat showed that changes in the components of cotyledons during storage may have contributed to the increased antioxidant activity of aged beans, regardless of seed coat color. These results revealed that roasting effectively improves the antioxidant activity of aged soybeans, regardless of seed coat color. We concluded that roasting is recommended for antioxidant properties, particularly regarding the effective use of aged beans.

## 1. Introduction

Soybeans are important foods because they retain high-quality nutrients and various functional components, such as polyphenols. The polyphenols in soybeans are mainly isoflavones with antioxidant properties. The seed coat of beans, which protects the cotyledon from the natural environment, contains many polyphenols that suppress oxidative damage in the cotyledon and the human body [1,2]. Various soybeans have different seed coat colors, such as yellow, green, brown, red, or black. Brown and black soybeans reportedly show higher radical scavenging activity than reddish-brown soybeans because of the rich amount of anthocyanins in the seed coat [3]. Beans are kept under ambient conditions, including high temperature and humidity, and their storage likely takes a long time. Such longterm storage affects the quality of beans. Concerning aged beans, there are no reports focusing on the effect of the storage of soybeans with different seed coat colors on antioxidant properties.

Common beans stored in adverse environments, such as high temperatures and humidity, are less likely to soften, even when cooked for a long time. This phenomenon is called the “hardening” or “hard-to-cook (HTC) defect”, which has been a serious problem in many countries where beans are staple foods [4,5]. The cause of HTC defects in common beans during storage is reportedly related to the insolubility of acidic and neutral pectin in the cell wall, cell wall ligninization, or suppression of starch swelling and gelatinization due to the denaturation of proteins around starch [6,7,8]. We previously confirmed that even soybeans, containing almost no starch, hardened when stored under high temperatures and humidity and reported that changes in cotyledon proteins are one of the causes of the hardening phenomenon [9,10]. Furthermore, many researchers have reported that the hardening of aged beans deteriorates palatability and nutrient inhibition, implying that effective use of aged beans should be developed. Moreover, hardened beans are often discarded, which wastes energy and resources and should be avoided.

In food processing, soybeans are sometimes roasted, ground into flour, and used as soybean flour with a good flavor. This flavor originates from the Maillard reaction products generated during food heating with high antioxidant activity. In various foods, such as sesame seeds, cashew nuts, and corn, the browning substances generated from the Maillard reaction are reportedly produced by roasting, with the antioxidant property increasing [11,12,13]. Thus, roasting is expected to improve the flavor and functionality of foods; the powderd form is easier to add to all kinds of dishes. Although studies on roasted soybeans have been conducted regarding browning reactions and antioxidant activity [14,15], none has focused on using aged beans effectively. In this study, three types of soybeans with different seed coat colors (yellow, blue, and black soybeans) were stored at high temperatures and high humidity (37 °C/75% RH), and their antioxidant activity before and after roasting was investigated. In addition, the influence of the seed coat on antioxidant activity was studied by removing the coat after roasting. Furthermore, we investigated the effect of storage and roasting on the angiotensin-converting enzyme (ACE) inhibitory activity of soybeans.

## 2. Materials and Methods

### 2.1. Soybean Samples

Three kinds of soybean (*Glycine max* L. Merrill) with different seed coat colors were used as samples. Yellow (Tsurunoko, Hokkaido, Japan), blue (Aobata, Yamagata Prefecture, Japan), and black soybeans (Hikarikuro, Hokkaido, Japan), harvested in 2019, were purchased from an online bean store. The general composition of soybeans with different seed coat colors was similar (Table 1). General ingredient analysis was performed according to the Standard Tables of Food Composition in Japan (2015, Seventh Revised Edition); the measurements were carried out by the Japan Functional Food Analysis and Research Center (Fukuoka, Japan).

### 2.2. Storage Conditions

The samples regarded as control beans were sealed in plastic bags and stored at 4 °C in a refrigerator until the experiment. Aged beans were stored at 37 °C and 75% RH for 60 days; a glass container filled with saturated saline solution was placed in a sealed container with the sample beans to maintain high humidity; the container was placed in a thermostatic chamber controlled at 37 °C. These hot and humid storage conditions cause soybeans to develop HTC defects [9,10].

### 2.3. Roasting Conditions

The beans were spread evenly on the baking sheet so that they did not overlap and were roasted in the gas oven. The roasting temperatures were 150, 170, 190, 210, and 230 °C; the heating time was uniformly 20 min.

### 2.4. Water Content of Roasted Soybean

The water content of soybeans was measured using a standard method (atmospheric drying). The samples were heated at 135 °C for 4 h and weighed until they reached a constant weight. The weight loss of the samples corresponded to the water content; the dry weight of each sample was determined.

### 2.5. Color Measurement

The color values were analyzed using a spectrophotometer (CM-700d, KONICA MI-NOLTA, Japan) represented by L*, a*, and b* according to the CIE color scale. L* represents the brightness from 0 (black) to 100 (white); the other two coordinates represent redness (+a*) to greenness (−a*) and yellowness (+b*) to blueness (−b*), respectively. The experiment was repeated three times. The hue change (ΔE) due to roasting was calculated using the following formula:

ΔE = [(L*after − L*before)^2^ + (a*after − a*before)^2^ + (b*after − b*before)^2^]^1/2^ (1)

### 2.6. Preparation for Soybean Extract

Samples used for the component analysis were ground into flour (Kawasaki Crusher DM-6) and passed through a No. 30 mesh screen. These processes were performed in a controlled room (4 °C) to avoid frictional heat effects. Approximately 1 g of soybean powder was added to 50 mL of 80% ethanol or 80% methanol solution and was shaken at room temperature (136 rpm, 26 °C, 60 min). The mixture was centrifuged (10,000 rpm, 4 °C, 15 min); the supernatant was filtered through a 0.45 μm membrane filter. The extracts were used to analyze the antioxidant activity and total phenol content.

### 2.7. Antioxidant Activity

Extracts obtained by 80% ethanol were used to analyze 1,1-diphenyl-2-pucrylhydrazy (DPPH) free radical scavenging activity and Oxygen Radical Absorbance capacity (ORAC).

DPPH’s free radical scavenging capacity was evaluated using the method described by Blois (1958) [16], with modifications. Ethanol (0.4 mL), 0.1 M acetate buffer of pH 5.5 (50 μL), and 0.5 mM DPPH reagent (100 μL) were added to 50 μL of the extracts; the mixed sample was left still in a dark place at 20 °C for 20 min. The absorbance was measured at 517 nm. DPPH values were expressed as micromoles of Trolox equivalents per gram of soybean (µmol TE/g) on a dry weight basis using the Trolox calibration curve. The experiment was repeated three times.

The ORAC assay was performed as described by Cao et al. [17] and Huang et al. [18], with modifications. The sample diluted with 75 mM phosphate buffer (25 μL) was placed in a black 96-well microplate, and 150 μL of 86.1 nM fluorescein was added; the mixture was incubated at 37 °C for 10 min. After incubation, 50 µL of 320 mM 2,2-azobis (2-amidinopropane) dihydrochloride (AAPH) solution was added to it; the absorbance at 760 nm was measured. The ORAC values were expressed as micromoles of Trolox equivalent per gram of soybean (µmol TE/g) on a dry weight basis, using the Trolox calibration curve. The linear range of the calibration curve was 5.0–50 µM. The experiment was re-peated three times.

### 2.8. Total Phenolic Content

The total phenolic content (TPC) was determined using the Folin–Ciocalteu method, with tannic acid as the standard. The extract obtained by 80% methanol as mentioned above (0.5 mL), distilled water (0.5 mL), 0.1 mL of 4-fold diluted Folin–Ciocalteu’s reagent solution, and 10% (*v*/*v*) Na_2_CO_3_ (0.2 mL) were mixed. After the mixture was allowed to stand at 26 °C for 60 min, the absorbance was measured at 760 nm. The sample concentration was obtained from a calibration curve using the standard; the results are expressed in mg per gram of dry weight. The experiment was repeated three times.

### 2.9. Assessed the ACE Inhibitory Activity

ACE inhibition activity was tested in 80% ethanolic extract. ACE inhibition activity test was conducted using ACE kit-WST (Dojindo, Japan). The assay procedure was in accordance with the technical manual provided with the assay kit. Briefly, ACE inhibition activity was tested by pipetting 20 μL sample solutions and 20 μL substrate buffers to the sample wells, then adding 20 μL of enzyme solutions and the mixtures were incubated at 37 °C for 60 min. Absorbance was measured at 450 nm using the microplate reader Synergy HTX (Biotek Instruments, Winooski, VT, USA). The ACE inhibitory activity of each extract was measured, and the 50% inhibitory concentration (IC_50_) was calculated. The experiment was repeated three times.

### 2.10. Statistical Analysis

Data are expressed as the mean ± standard deviation. The significance of differences between control and each treatment was analyzed using Student–Newman–Keuls test. Data analysis was subjected to multiple comparison tests (*p* < 0.05) using SPSS Statistics version 25.0 software for Windows (IBM, Armonk, NY, USA).

## 3. Results and Discussion

### 3.1. Color Characteristic of Roasted Soybean

As shown in Table 2, the color values of the roasted soybeans changed with increasing temperature, particularly at above 190 °C; the change tendency was similar among the three kinds of soybeans. The L* value of all beans decreased significantly at roasting temperatures above 190 °C, indicating that the seed coat color became darker regardless of the original color. The color difference ΔE values, which express the overall color change, increased with increasing roasting temperature for all beans. The values of yellow soybeans were the highest at temperatures above 190 °C, suggesting that the thinner the seed coat color, the clearer the color change. The decrease in the L* value, with increasing a* and b* values during roasting, is reportedly caused by the brown pigment, which is the product of the Maillard reaction [15,19], consistent with our results. The color changes of soybeans by roasting were due to non-enzymatic browning associated with the Maillard reaction. When roasted at 230 °C, all the color values changed drastically; all the roasted samples appeared undesirable. From these results, 190 °C was considered the optimal roasting temperature.

### 3.2. Antioxidant Activity and TPC of Soybean

**Figure 1:**
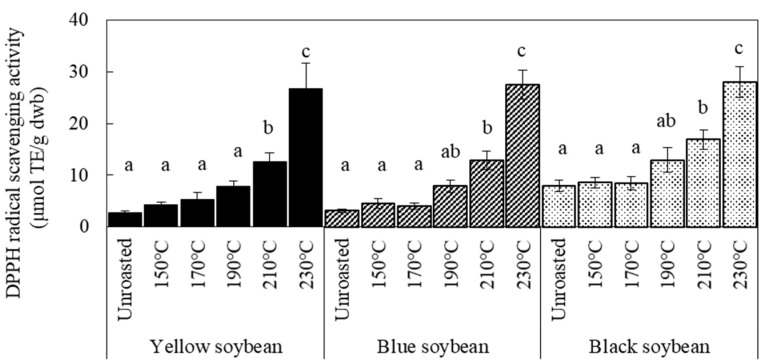
Effect of roasting temperatures on DPPH radical scavenging activity. Sample soybeans were stored at 4 °C. Significant differences within the same bean are indicated by lowercase letters a, b, and c. *p* < 0.05. *n* = 3.

**Figure 2:**
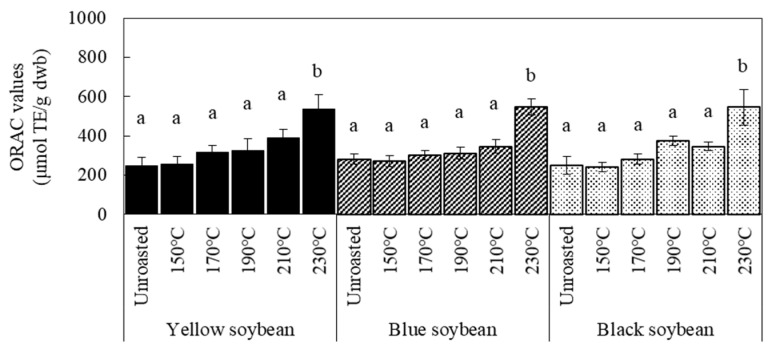
Effect of roasting temperatures on ORAC values of soybeans. Sample soybeans were stored at 4 °C. Significant differences within the same bean are indicated by lowercase letters a and b. *p* < 0.05. *n* = 3.

The changes in DPPH and ORAC values of soybeans with increasing roasting temperature are shown in Figure 1 and Figure 2, respectively. Sample soybeans were stored at 4 °C. The DPPH radical scavenging activity before roasting was highest in black soybeans. These results indicate that black soybean with dark-colored seed coats had higher antioxidant activity than yellow soybean with light-colored seed coats, indicating the effect of pigment in the coat, consistent with previous studies [2,14]. After roasting, the DPPH values increased at temperatures above 190 °C for the three kinds of soybean; however, yellow soybeans showed a small increase at 170 °C. Notably, the degree of increase in DPPH values for all beans by roasting was almost similar at each temperature; the difference in the value between raw and roasted soybeans was approximately 5 μmol TE/g at 190 °C, 9.0–9.9 μmol TE/g at 210 °C, and 20–22 μmol TE/g at 230 °C. These results indicate that the increased antioxidant activity of the three kinds of soybeans after roasting was caused by some components in the cotyledon. The ORAC values significantly increased when roasted at 230 °C.

**Figure 3:**
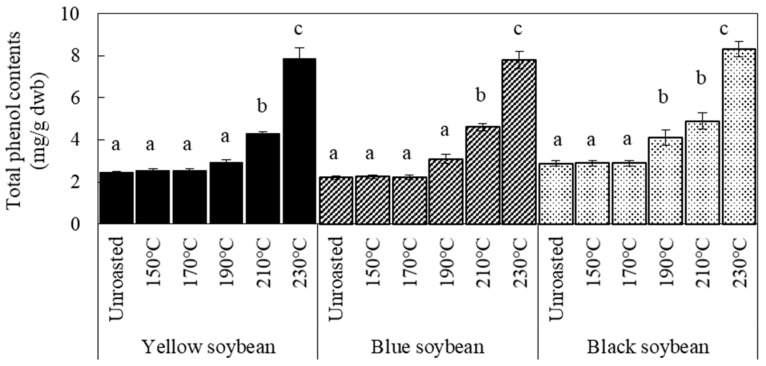
Effect of roasting temperatures on total phenol contents of soybean. Sample soybeans were stored at 4 °C. Significant differences within the same bean are indicated by lowercase letters a, b, and c. *p* < 0.05. *n* = 3.

The TPC of the soybeans before and after roasting is shown in Figure 3. The increasing trend in TPC is similar to that of the DPPH values (Figure 1). These results suggest that the increased TPC due to roasting was unaffected by the seed coat color. Many studies concerning roasted beans have reported a high positive correlation between TPC and antioxidant capacity [19,20,21]. Moreover, a positive correlation between color changes due to non-enzymatic browning and TPC can be found in many foods, including beans [11,12]. Our data showed a robust relationship among the measured values. As shown in Table 3, almost all the determination coefficients of the linear regression analysis in the combination of the two among browning, antioxidant activity, and TPC of each soybean were over 0.9, indicating a high positive correlation. These results suggest that browning and phenolic compounds produced by roasting increase the antioxidant activity of soybeans.

The Maillard reaction is a chemical reaction between nitrogen compounds, such as amino acids, and carbonyl compounds, such as reducing sugars, during the heat processing of food, producing brown substances and distinctive flavors. Melanoidin, a brown pigment formed in the late stages of the Maillard reaction, has reducing and antioxidant properties [22]. Furthermore, thermal processing disrupts cell membranes and walls, releasing soluble phenolic contents from insoluble ester bonds [13]. Rongrong et al. (2017) [14] reported that roasting processes alter phenolic compounds through the Maillard reaction, resulting in increased antioxidant potential due to the breakdown of insoluble phenolic compounds. Furthermore, according to a previous study on maize bran, an increased amount of solubilized ferulic acid increases total antioxidant activity; significant solubilization occurs only at temperatures higher than 180 °C [23]. Hyo et al. [21] reported that roasting Korean black soybeans at different temperatures increased the DPPH radical scavenging activity by 2.24% at 150 °C and 66.88% at 250 °C. In addition, they showed that Maillard reaction products and phenolic compounds solubilized during roasting were related to the increase in the DPPH values.

Therefore, we proposed that the increase in antioxidant activity by roasting is related to Maillard reaction products and phenols produced in the cotyledons rather than in the seed coat. In addition, temperatures of ≥190 °C were needed to increase the antioxidant activity. Although this activity was the highest at 230 °C, 190 °C was regarded as the appropriate roasting temperature because the color of roasted soybeans was too dark, as mentioned in Table 2.

### 3.3. Color Characteristic of Aged Soybeans

The effects of soybean storage and roasting on color values are shown in Table 4. All aged beans showed lower L* values and higher a* values than control beans. The ΔE values were 4.0 for yellow soybeans, 8.8 for blue soybeans, and 6.6 for black soybeans. The value of ΔE needs to be >3 to differentiate the color difference with the naked eye. These results indicate that the seed coat becomes darker during storage, which can be visually recognized. Moreover, yellow soybeans stored under high temperatures and high humidity reportedly have a dark yellow color owing to changes in lipids during storage [24]. This phenomenon indicates that soybeans turned brown because of the high temperature and humidity without heating. Table 4 shows that when the aged beans were roasted, the L* values decreased markedly compared to after storage. However, the differences before and after roasting the aged beans were almost similar to those without storage (Table 2). Therefore, it is suggested that the causes of the color change of beans by storage differ from those of beans by roasting.

### 3.4. Antioxidant Activity and TPC due to Roasting in Aged Soybeans

**Figure 4:**
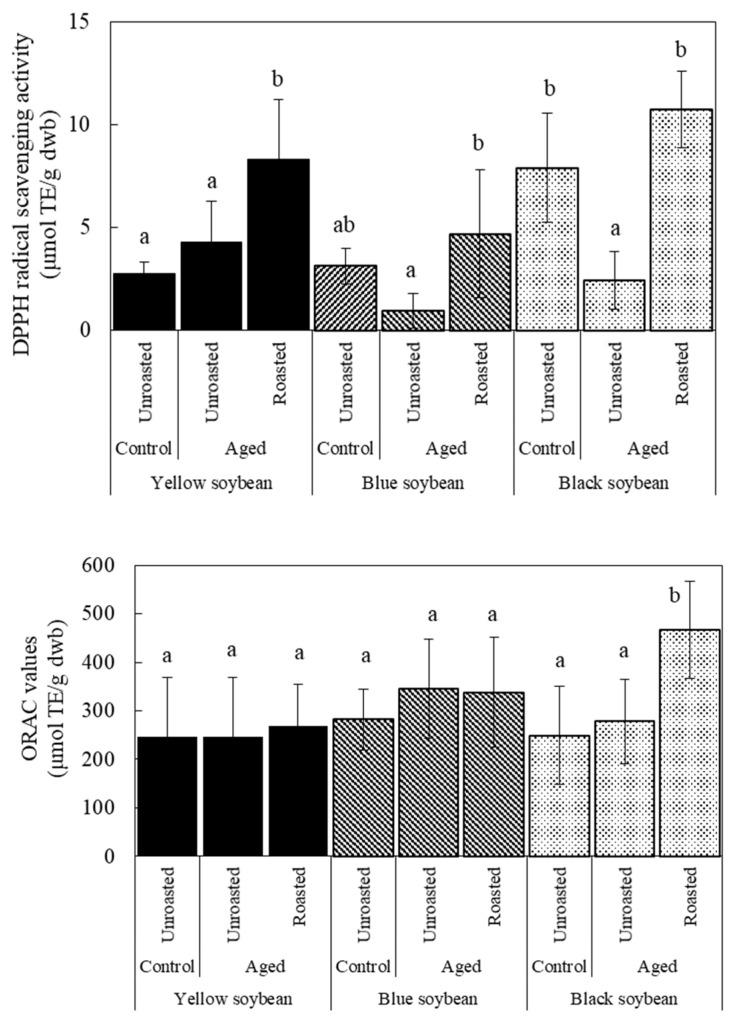
DPPH radical scavenging activity and ORAC values of soybeans affected by storage and roasting. Control were stored at 4 °C/80% RH for 60 days; Aged beans were stored at 30 °C/75% RH for 60 days. Roasting condition was 190 °C for 20 min. Significant differences within the same bean are indicated by lowercase letters a and b. *p* < 0.05. *n* = 3.

Figure 4 shows the DPPH radical scavenging activity and ORAC values of the aged beans before and after roasting. Compared to the no-storage conditions (Figure 2), yellow soybeans had the highest antioxidant activity. However, the effect of storage at high temperatures and humidity on the DPPH value varied among the three kinds of soybeans. A small increase in DPPH values was observed for yellow soybeans, whereas a decrease was observed for blue and black beans. In contrast, when roasted at 190 °C, DPPH values of all the aged beans significantly increased regardless of the seed coat color. Yellow soybeans stored at high temperatures and high humidity reportedly changed the protein content in the cotyledon [9,25]. Therefore, it was suggested that some decompositions due to enzymes occurred during storage, and the products were related to the increased antioxidant properties of the aged beans when roasted. Consequently, we hypothesized that changes in the composition of the cotyledons during storage have a large effect on the antioxidant activity of aged soybeans compared to that of the seed coat. Concerning ORAC values of aged beans, there were no changes by storage and roasting, except for black soybeans, whose values increased only when roasted. Concerning black soybeans, the degree of decreased DPPH values under storage and increased values by roasting was the largest among the three, suggesting the existence of some causes. These results show that the depleted antioxidant activity of aged beans due to storage could be improved by roasting for more effective utilization.

**Figure 5:**
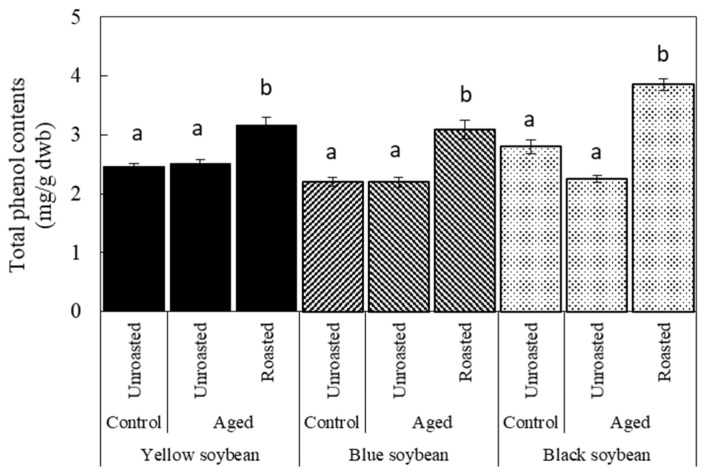
Total phenol contents of soybeans affected by storage and roasting. Storage condition: Control were stored at 4 °C/80% RH for 60 days; Aged beans were stored at 30 °C/75% RH for 60 days. Roasting condition was 190 °C for 20 min. Significant differences within the same bean are indicated by lowercase letters a and b. *p* < 0.05. *n* = 3.

The effects of storage and roasting on TPC are shown in Figure 5. No significant difference was observed between the control and aged beans in the unroasted state. However, TPC increased significantly after roasting, with the highest increase observed in black soybeans. These changes in TPC did not show a similar trend as those in DPPH radical scavenging activity. Therefore, we speculated that components other than phenolic com-pounds contribute to the change in antioxidant activity during storage. In addition, the components, including phenolic compounds, contribute to increased antioxidant activity after roasting. Machado et al. [26] reported that, regarding white, red, and black common beans with different seed coat colors, HTC beans had decreased antioxidant activity and TPC with a high correlation between them. However, the characteristics of aged soybeans differed from those of common beans. Although further research is needed to clarify the mechanism of the change in antioxidant properties by storage and roasting, roasting is a useful treatment for improving the antioxidant properties of aged beans.

### 3.5. Antioxidant Activity with or without Seed Coat

**Figure 6:**
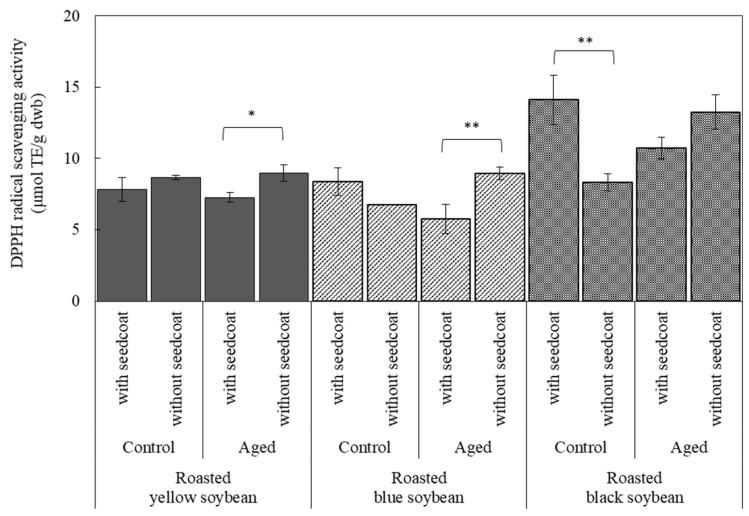
Effect of seed coat on DPPH radical scavenging activity of roasted soybeans before and after storage. Storage condition: Control were kept in a refrigerator (4 °C) for 60 days; Aged beans were stored at 30 °C/75% RH for 60 days. Roasting condition was 190 °C for 20 min. * indicates that there is significant difference between with seed coat and without seed coat in the same beans. * *p* < 0.05, ** *p* < 0.01. *n* = 6.

To investigate the effect of seed coats on antioxidant activity, the seed coats of the roasted beans were removed, and the DPPH values were compared with those of the beans with seed coats. However, the seed coat of the raw beans could not be removed because they were firmly attached to the cotyledon. The results are shown in Figure 6. Concerning the control beans before storage, no significant difference in DPPH values with and without seed coat was observed in yellow and blue soybeans. In contrast, a significant decrease in DPPH values was observed in black soybeans, confirming the effect of the pigment in the seed coat of black beans before storage was confirmed.

Conversely, when aged beans were roasted at 190 °C, the DPPH radical scavenging activity of all the aged beans without seed coats was significantly higher than that of those with seed coats. DPPH values are unaffected by removing the seed coat if the compositions related to the antioxidant activity are distributed equally in the seed coat and cotyledon. However, as seen in Figure 6, the DPPH values of all the aged beans without seed coats were higher than those of the beans with seed coats, suggesting that the component contained in the cotyledon of the aged beans contributed more to the antioxidant activity than that of the seed coat. Considering that there was no difference between the beans with and without seed coat concerning control beans of yellow and blue soybeans, these beans were not influenced by the seed coat. However, regarding control beans of black soybeans, the DPPH values of the beans without seed coat decreased because the effect of the dark-colored seed coat on the values was significant.

These results revealed that roasting increases the antioxidant activity of aged beans due to changes in the cotyledon’s components during storage, even if storage caused a decrease in antioxidant activity.

### 3.6. ACE Inhibition Activity

Finally, we examined the ACE-inhibitory activity of soybeans. Peptides obtained from fermented or enzymatic hydrolysates of soy proteins have been reported to have ACE inhibitory activity [27]. ACE is an angiotensin I-converting enzyme; foods with ACE inhibitory activity are widely used to prevent hypertension [25]. However, no reports have examined the changes in ACE inhibitory activity in aged soybeans. The results are shown in Table 5. The IC_50_ values indicate that the smaller the value, the higher the ACE inhibitory activity. The IC_50_ values of aged beans were significantly lower than those of control beans before roasting. Although there was a small decrease in IC_50_ for both control and aged beans after roasting, the high values of all aged beans were almost maintained. These findings suggest that ACE inhibitory activity increases when soybeans are stored at high temperatures and humidity, regardless of the seed coat color.

Betancur-Ancona et al. [25] reported that amino acids exhibiting high ACE inhibitory activity in aged common beans might be hydrophobic amino acids, aromatic amino acids, or branched amino acids and that short peptides are more effective in inhibiting ACE than long peptides. In other words, a higher ACE inhibitory activity can be expected by hydrolyzing proteins to produce smaller peptides. In this study, aged soybeans showed higher ACE inhibitory activity than control beans, probably because proteases acted inside the cotyledons during storage and degraded them into highly active peptides. The small decrease in ACE inhibitory activity by roasting might be caused by both the Maillard reaction and the decomposition of the components of the cotyledon.

Besides the antioxidant properties, an increase in ACE inhibitory activity after soybean storage was observed, and the activity was almost maintained even after roasting. These findings suggest that roasting aged beans are recommended from the viewpoint of nutrients and functional properties.

## 4. Conclusions

We investigated the effects of storing and roasting soybeans on their antioxidant properties, using aged beans that caused hardening in an HTC state. Three kinds of soybeans with different seed coat colors were roasted at 150–230 °C for 20 min. Consequently, DPPH radical scavenging activity and ORAC value similarly increased at temperatures above 190 °C. Concerning raw beans, black soybeans had the highest antioxidant activity, indicating the effect of pigments in the seed coat. Concerning aged beans stored at 37 °C/75% RH for 60 days, DPPH radical scavenging activity increased in yellow soybeans and decreased in green and black soybeans. Conversely, when roasted at 190 °C for 20 min, the antioxidant activity of all the aged beans significantly increased.

The analysis of roasted beans with and without seed coat suggested that changes in some components of the cotyledon during storage contributed to the increased antioxidant activity of aged beans by roasting, regardless of the seed coat color. These results revealed that roasting effectively improves the antioxidant activity of aged beans. In addition, we found that three kinds of soybean showed increased ACE inhibitory activity during stor-age; their values were nearly maintained even after roasting. We concluded that roasting beans is recommended for improved antioxidant activities, particularly regarding the effective use of aged beans.

## Figures and Tables

**Table 1:** General ingredients of three kinds of soybean.

	Energy	Water	Protein	Lipid	Carbohydrate ^1^	Ash	Na
	kcal/100 g	g/100 g	g/100 g	g/100 g	g/100 g	g/100 g	mg/100 g
Yellow soybean	411	13.5	32.4	16.7	32.8	4.6	2
Blue soybean	419	13.1	34.8	17.7	30	4.4	1
Black soybean	417	13.6	31.2	17.9	32.8	4.5	2

The general ingredient analysis was a method according to Standard Tables of Food Composition in Japan (2015, Seventh Revised Edition). ^1^ Carbohydrate value was an amount deducted a sum of “protein” “lipid” and “ash” from total dried weight vs.

**Table 2:** Changes in color values of soybeans by roasting at various temperatures.

	Roasting Temperature (℃)		L*			a*			b*			ΔE	
Yellow soybean	Unroasted	89.9	±	1.1	−3.3	±	0.1	25.8	±	0.7			
	150	80.4	±	3.2*	2.9	±	1.8*	27.8	±	3.3	11.5	±	4.0
	170	78.6	±	2.1*	5.3	±	1.3*	30.8	±	1.0*	15.0	±	2.3
	190	65.0	±	4.3*	8.8	±	0.8**	32.3	±	2.1*	28.4	±	4.7
	210	47.0	±	2.0**	11.2	±	0.6**	29.9	±	1.9*	45.5	±	3.1
	230	30.1	±	3.6**	9.7	±	2.3**	18.5	±	7.8*	61.6	±	10.4
Blue soybean	Unroasted	78.2	±	5.4	−5.5	±	3.5	25.6	±	2.3			
	150	78.4	±	2.7	0.1	±	1.0	29.7	±	1.5	7.0	±	3.1
	170	72.7	±	3.3	5.5	±	0.9	34.1	±	0.8	14.9	±	1.8
	190	56.5	±	2.8**	9.9	±	1.4**	32.0	±	3.5*	27.3	±	9.4
	210	45.3	±	4.5**	11.8	±	1.4**	27.7	±	5.8	37.2	±	9.5
	230	28.3	±	4.8**	8.2	±	3.0**	12.7	±	7.3*	53.3	±	12.6
Black soybean	Unroasted	76.1	±	5.0	−1.7	±	1.6	21.9	±	1.7			
	150	77.9	±	0.3	0.7	±	0.6	23.2	±	1.7	3.0	±	2.0
	170	70.0	±	1.0*	5.9	±	0.4*	30.3	±	1.2*	14.5	±	2.7
	190	57.2	±	9.2	10.0	±	2.4*	31.5	±	2.2*	26.5	±	8.0
	210	49.2	±	4.3*	10.5	±	1.0***	28.6	±	2.5*	32.8	±	6.6
	230	23.3	±	7.2**	7.8	±	2.1**	12.1	±	4.5*	57.2	±	12.1

Sample soybeans were stored at 4 °C. L* represents brightness from 0 (black) to 100 (white). The other two coordinates represent redness (+a*) to greenness (−a*), and yellowness (+b*) to blueness (−b*), respectively. The hue change (ΔE) due to roasting was calculated by the following formula. ΔE = [(L*after − L*before)^2^ + (a*after − a*before)^2^ + (b*after − b*before)^2^]^1/2^. Values are indicated as the mean ± SD (*n* = 6). * *p* < 0.05, ** *p* < 0.01, *** *p* < 0.001 compared with the unroasted group. Roasting time: 20 min for each temperature.

**Table 3:** Determination coefficients of linear regression analysis in combination of the two among antioxidant activity, total phenol content and color values.

		ORAC Values	Total Polyphenol Content	L^*^	ΔE
Yellow soybean	DPPH radical scavenging activity	0.961	0.980	0.935	0.936
	ORAC values	-	0.932	0.915	0.911
Blue soybean	DPPH radical scavenging activity	0.968	0.981	0.962	0.969
	ORAC values	-	0.977	0.895	0.905
Black soybean	DPPH radical scavenging activity	0.908	0.971	0.979	0.974
	ORAC values	-	0.932	0.940	0.945

**Table 4:** Changes in color values of soybeans by storage and roasting.

				L*			a*			b*			ΔE	
Yellow soybean	Control	Unroasted	89.9	±	1.1	−3.3	±	0.1	25.8	±	0.7			
	Aged	Unroasted	85.9	±	0.8	−1.0	±	1.5	24.8	±	1.3	4.0	±	0.6
		Roasted	62.8	±	6.9	10.2	±	3.4	31.2	±	2.1	30.8	±	7.1
Blue soybean	Control	Unroasted	78.2	±	5.4	−5.5	±	3.5	25.6	±	2.3			
	Aged	Unroasted	74.3	±	5.1	−2.0	±	1.7	26.7	±	4.1	8.8	±	2.1
		Roasted	55.7	±	7.1	12.4	±	1.7	28.8	±	2.8	29.0	±	8.0
Black soybean	Control	Unroasted	76.1	±	5.0	−1.7	±	1.6	21.9	±	1.7			
	Aged	Unroasted	73.5	±	3.3	2.4	±	1.8	24.8	±	3.4	6.6	±	4.3
		Roasted	55.3	±	3.2	12.1	±	1.5	28.0	±	3.5	25.9	±	7.4

Storage condition: Control were stored at 4 °C/80% RH for 60 days; Aged beans were stored at 30 °C/75% RH for 60 days. Roasting condition was 190 °C for 20 min. L* represents brightness from 0 (black) to 100 (white). The other two coordinates represent redness (+a*) to greenness (−a*), and yellowness (+b*) to blueness (−b*), respectively. The hue change (ΔE) due to roasting was calculated by the following formula. ΔE = [(L*after − L*before)^2^ + (a*after − a*before)^2^ + (b*after − b*before)^2^]^1/2^.

**Table 5:** ACE inhibitory activity of soybeans affected by storage and roasting.

		Yellow Soybean						Blue Soybean						Black Soybean					
		Control			Aged			Control			Aged			Control			Aged		
ACE inhibitation rate (%)	Untreated	30.0	±	5.4 ^a^	59.2	±	2.9 ^b^	54.2	±	14.2 ^a^	55.3	±	9.3 ^a^	57.1	±	8.2 ^a^	77.3	±	4.4 ^b^
	Roasted	41.0	±	10.9 ^a^	52.4	±	7.8 ^b^	49.8	±	10.6 ^a^	41.0	±	7.8 ^b^	55.1	±	13.7 ^a^	62.3	±	5.1 ^a^
IC_50_ (mg/mL)	Untreated	2.72			0.57			3.13			1.45			0.72				nd	
	Roasted	2.73			0.82			3.24			1.68			1.90			0.65		

Mean ± SD, Significant differences within the same bean are indicated by lowercase letters a and b. *p* < 0.05. *n*=3.

## Data Availability

The data are available from the corresponding author.
